# Establishing a Health CASCADE–Curated Open-Access Database to Consolidate Knowledge About Co-Creation: Novel Artificial Intelligence–Assisted Methodology Based on Systematic Reviews

**DOI:** 10.2196/45059

**Published:** 2023-07-18

**Authors:** Danielle Marie Agnello, Quentin Emile Armand Loisel, Qingfan An, George Balaskas, Rabab Chrifou, Philippa Dall, Janneke de Boer, Lea Rahel Delfmann, Maria Giné-Garriga, Kunshan Goh, Giuliana Raffaella Longworth, Katrina Messiha, Lauren McCaffrey, Niamh Smith, Artur Steiner, Mira Vogelsang, Sebastien Chastin

**Affiliations:** 1 School of Health and Life Sciences Glasgow Caledonian University Glasgow United Kingdom; 2 Department of Community Medicine and Rehabilitation Umeå University Umeå Sweden; 3 Institute of Informatics and Telecommunications National Centre of Scientific Research Demokritos Athens Greece; 4 Department of Digital Systems University of Piraeus Piraeus Greece; 5 Department of Public Health and Primary Care Ghent University Ghent Belgium; 6 Department of Movement and Sports Sciences Ghent University Ghent Belgium; 7 Department of Sport Sciences Faculty of Psychology Education and Sport Sciences Blanquerna - Ramon Llull University Barcelona Spain; 8 Department of Physical Therapy Faculty of Health Sciences Blanquerna - Ramon Llull University Barcelona Spain; 9 Department of Public and Occupational Health Amsterdam Public Health Research Institute, Amsterdam University Medical Centers Vrije Universiteit Amsterdam Amsterdam Netherlands; 10 Yunus Centre Glasgow Caledonian University Glasgow United Kingdom

**Keywords:** co-creation, co-production, co-design, database, participatory, methodology, artificial intelligence

## Abstract

**Background:**

Co-creation is an approach that aims to democratize research and bridge the gap between research and practice, but the potential fragmentation of knowledge about co-creation has hindered progress. A comprehensive database of published literature from multidisciplinary sources can address this fragmentation through the integration of diverse perspectives, identification and dissemination of best practices, and increase clarity about co-creation. However, two considerable challenges exist. First, there is uncertainty about co-creation terminology, making it difficult to identify relevant literature. Second, the exponential growth of scientific publications has led to an overwhelming amount of literature that surpasses the human capacity for a comprehensive review. These challenges hinder progress in co-creation research and underscore the need for a novel methodology to consolidate and investigate the literature.

**Objective:**

This study aimed to synthesize knowledge about co-creation across various fields through the development and application of an artificial intelligence (AI)–assisted selection process. The ultimate goal of this database was to provide stakeholders interested in co-creation with relevant literature.

**Methods:**

We created a novel methodology for establishing a curated database. To accommodate the variation in terminology, we used a broad definition of co-creation that encompassed the essence of existing definitions. To filter out irrelevant information, an AI-assisted selection process was used. In addition, we conducted bibliometric analyses and quality control procedures to assess content and accuracy. Overall, this approach allowed us to develop a robust and reliable database that serves as a valuable resource for stakeholders interested in co-creation.

**Results:**

The final version of the database included 13,501 papers, which are indexed in Zenodo and accessible in an open-access downloadable format. The quality assessment revealed that 20.3% (140/688) of the database likely contained irrelevant material, whereas the methodology captured 91% (58/64) of the relevant literature. *Participatory* and variations of the term *co-creation* were the most frequent terms in the title and abstracts of included literature. The predominant source journals included health sciences, sustainability, environmental sciences, medical research, and health services research.

**Conclusions:**

This study produced a high-quality, open-access database about co-creation. The study demonstrates that it is possible to perform a systematic review selection process on a fragmented concept using human-AI collaboration. Our unified concept of co-creation includes the co-approaches (co-creation, co-design, and co-production), forms of participatory research, and user involvement. Our analysis of authorship, citations, and source landscape highlights the potential lack of collaboration among co-creation researchers and underscores the need for future investigation into the different research methodologies. The database provides a resource for relevant literature and can support rapid literature reviews about co-creation. It also offers clarity about the current co-creation landscape and helps to address barriers that researchers may face when seeking evidence about co-creation.

## Introduction

### Background

Co-creation emerges from the participatory research paradigm, is grounded in a history of community engagement, and was first documented in the 1970s [1]. According to Greenhalgh et al [2], co-creation is a promising strategy to increase the effectiveness and impact of health interventions and claims to address complex problems in public health (eg, the obesity epidemic, persisting poverty, and food insecurity), which are particularly resistant to resolution, specifically in vulnerable populations [2,3]. Co-creation also responds to the growing demand from the public to be involved in research aimed at ensuring health interventions are better tailored to their needs and circumstances [2]. Furthermore, the explicit reference to co-creation in many fields of science and sectors has grown exponentially in recent years, and it is now expected, and often demanded, by funders, governments, and policy makers to democratize and accelerate the impact of research and innovation [4].

Co-creation aims to engage stakeholders from all the nodes of the quadruple helix (academia, industry, government, and users) to create collectively effective and sustainable solutions. However, without an evidence base or a robust and trustworthy methodology, using co-creation can remain a blind praxis [5]. Co-creation is resource intensive and, if conducted poorly, can waste time and resources without clear impacts and outputs. There is a real danger that this will ultimately lead to growing distrust toward co-creation from researchers, the public, policy makers, and other key stakeholders. More importantly, it might put the public at risk of detrimental effects and potential unintended consequences (sensitive information leaked, ethical misconduct, etc) from poorly evidenced and poorly conducted co-creation [4].

Presently, the progress of co-creation in public health is hindered by the limited availability of comprehensive and integrated literature and empirical evidence, further complicated by the absence of a standardized methodology. Assembling a database of published literature from multidisciplinary sources is a key step toward facilitating the integration of diverse perspectives and enabling the identification and dissemination of best practices across multiple fields of research. However, there are two considerable challenges to achieving this. First, there is a lack of clarity about co-creation, including the interchangeable use of terminology when referring to co-creation. Second, the rapid increase in scientific publishing means that obtaining exhaustive and comprehensive literature requires dealing with a vast body of information, making it almost impossible to complete it on time within the standards for quality research. For instance, by the time the research is completed, it may already be outdated [6].

### Fragmentation of Knowledge About Co-Creation

Bauman [7] and Iwarsson et al [8] highlight the interchangeable use of the terms *co-creation*, *co-design*, *co-production*, *participatory research*, *patient and public involvement*, and *collaborative research* when describing a development process involving multiple stakeholders. Vargas et al [9] argued that co-creation is considered an overarching principle that includes both co-design and co-production. Co-creation has been used in different fields, for example, it can be perceived as user involvement in designing goods, as well as codelivery of public services [9]. In addition, Masterson et al [10] recently uncovered >500 different definitions of co-production and co-design, and the rate of introducing new definitions is increasing over the past decade.

The use of interchangeable terms and varying definitions in co-creation research has led to potential conceptual fragmentation [11]. Messiha et al [12] recently discussed this issue when investigating systematic theory building, highlighting the risk of knowledge fragmentation when inconsistent terminologies related to co-creation are used. Furthermore, Gray et al [13] discussed the work by Hirsch and Levin [14] in characterizing the fragmentation process in organizational sciences and highlighting the life cycle of emerging ideas. When research emphasizes differences between aspects of a central idea, it can lead to divergence, resulting in nonoverlapping ideas and an increasing prevalence of contextualizing research, ultimately leading to fragmentation [13]. Consequently, fragmentation and poor communication can impede scientific progress and undermine the original concept [13,15]. Although it is encouraging that many stakeholders recognize the value of co-creation, fragmentation hinders knowledge sharing and the effective application of co-creation in practice. Therefore, a unified concept of co-creation is necessary to address the complexity arising from the broad and interchangeable use of the terminology.

Given the current situation, it is increasingly challenging to gain a comprehensive overview of co-creation or to gather appropriate theoretical and methodological support in the literature [16]. This is compounded by the fact that in recent years, the overall scientific publication output has been increasing at an annual rate of 3% from 1980 to 2012, and the volume of publications doubles every 24 years [17,18]. Owing to this rate of increase, it is becoming difficult, if not impossible, to approach this vast amount of literature without having to make compromises in keywords and database selection [19]. Unfortunately, these concessions can jeopardize the quality of the research as well as decrease the possibility of gaining a comprehensive and exhaustive evidence base. To manage the potential fragmentation, an overinclusive approach to co-creation terms is needed in addition to an expanded systematic review process that includes an artificial intelligence (AI)–assisted selection process to manage the large volume of literature resulting from such an approach.

### Objective

This study aimed to synthesize knowledge about co-creation across various fields through the development and application of an AI-assisted selection process. The ultimate goal of this database is to provide stakeholders interested in co-creation with relevant literature.

## Methods

### Overview

We developed a novel selection methodology using AI to include all relevant literature. To ensure the quality of our methodology, we based it on established frameworks such as the Cochrane Collaboration and PRISMA (Preferred Reporting Items for Systematic Reviews and Meta-Analyses) [20]. Systematic reviews have traditionally used meticulously designed search strategies that use various combinations of precise terms to identify pertinent literature and reduce the need for extensive screening. However, the interchangeable use of co-creation terminology necessitated a broad search strategy to avoid the inadvertent exclusion of relevant literature. This broad search strategy resulted in a vast amount of literature.

To handle the search results, we used a set of selection criteria aiming to capture key aspects of co-creation. In addition, we used ASReview (Open Science Lab), an AI software [21], to assist a multidisciplinary team of researchers in efficiently excluding irrelevant literature during the selection process. This AI software allowed us to reduce the volume of papers requiring screening, whereas the researchers helped to prevent terminological bias and ensure a broad consensual conceptual understanding of co-creation. As the objective of this study was to identify and consolidate relevant literature about co-creation, only the conventional systematic review steps up to the selection process were followed [20], and no protocol was registered.

### Search Strategy

To ensure a comprehensive search strategy, we compiled an exhaustive list of keywords that could represent co-creation in a broad sense [10]. The list was refined through rounds of discussions with a multidisciplinary group of co-creation researchers, resulting in the final selection of keywords. The final set of keywords in English included co-creat*, *co-conception*, *co-production*, *public and patient involvement*, *public participation*, *participatory*, *experience-based design*, *co-design*, *user involvement*, *collaborative design*, and *citizen science*. The team searched PubMed, CINAHL, and all 47 databases within ProQuest with tailored search strategies. Details on the search strategy and results are provided in Multimedia Appendix 1.

All the references retrieved from the search were downloaded in Microsoft Excel format and merged into 1 document (duplicates were removed). Any article that did not contain an abstract was removed and archived for manual screening. The research team identified 10 relevant and 5 irrelevant articles to provide preliminary material to ASReview [22] to begin its learning process [21]. Owing to the potential duplicates and to reduce the risk of overfitting, the full set of literature was randomized before the selection process began [23].

### Selection Process

A total of 14 researchers affiliated with the Health CASCADE Marie Skłodowska-Curie Innovative Training Network [24] were enlisted to conduct the selection process using ASReview [21]. The researchers were paired and performed double-blind screening on the same set of literature. However, it is important to note that because ASReview reacts to each decision of the researchers, the pairs may not have screened the same set of literature before reaching the stop rules (these rules are described below).

**Figure 1:**
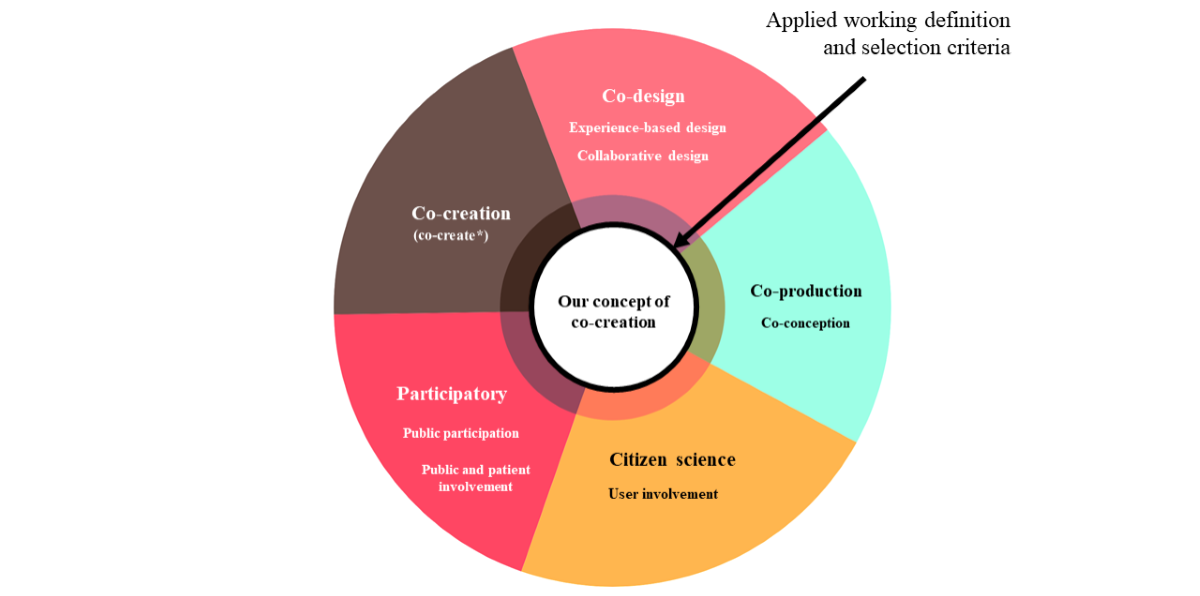
Screening with a unified concept of co-creation.

We used a multidisciplinary and AI-assisted approach to establish a unified concept of co-creation, despite the interchangeable use of terms in the literature. This allowed us to assemble a set of literature that comprehensively represented co-creation, regardless of the intended meaning of specific terms (Figure 1).

The selection criteria are presented in Textbox 1. The selection criteria included a new definition of co-creation that captured the essence of existing definitions and was inclusive of diverse fields while still accommodating the variation in terminology. Co-creation is defined as any act of collective creativity that involves a broad range of relevant and affected actors in creative problem-solving that aims to produce a desired outcome [25,26].

In the selection process, researchers screened records presented by ASReview until they reached the 2 stop rules: they had screened 10% (1440/14,393) of their packet of papers and they found 100 irrelevant papers in a row. Papers without an abstract were not included in the ASReview screening. Instead, they were evaluated manually by the researchers using the same criteria for selection [27].

**Inclusion and exclusion criteria for the selection process.**

**Inclusion criteria**

- Includes at least 1 or more of the keywords in the title or abstract of the article.
- On the basis of the abstract, the study adheres to the working definition of co-creation.
- The title and abstract are both written in English.

**Exclusion criteria**

- The title or abstract did not contain any of the keywords.
- On the basis of the abstract, the study did not adhere to the working definition of co-creation.
- The title or abstract is written in a language other than English.

### Data Consolidation

**Figure 2:**
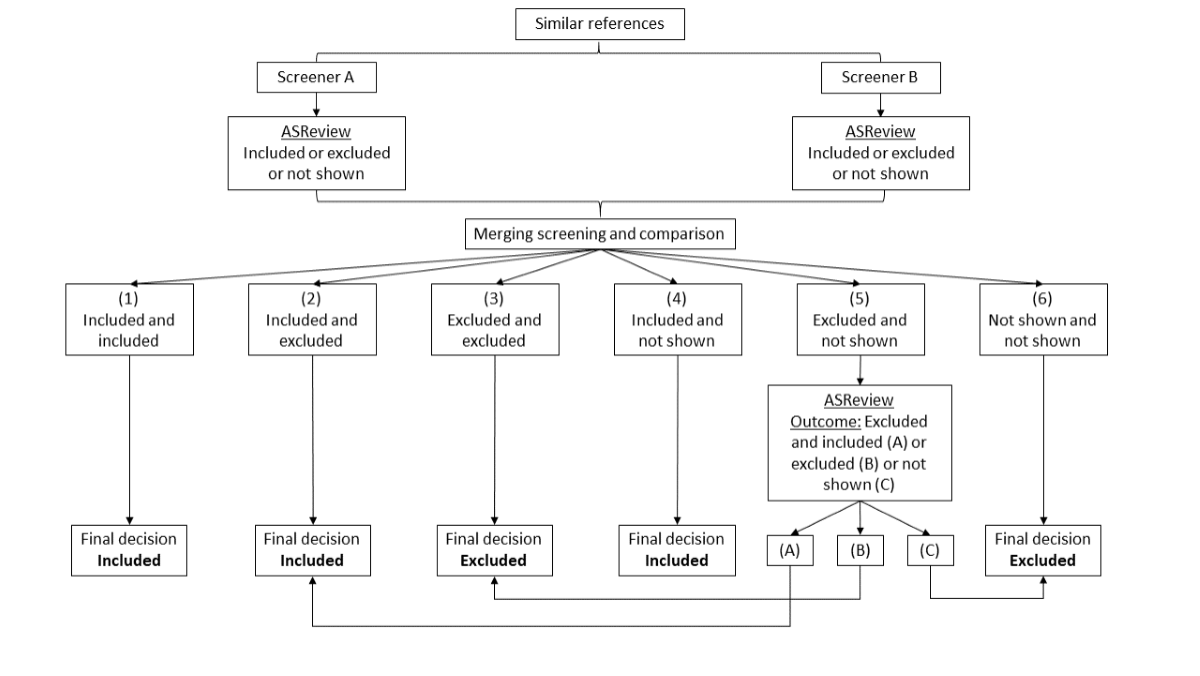
Decision support tree for double screening of papers.

During the selection process in ASReview, three possible outcomes were possible: (1) a paper was included, (2) a paper was excluded, or (3) the paper was not shown to the researcher (meaning ASReview did not show the reference to the researcher before the stop rule was reached). Therefore, during the screening step, each paper received a decision, creating 6 possible combinations. These combinations are depicted in Figure 2.

Figure 2 visualizes the decision support tree applied during the selection process. As our screening strategy prioritized overinclusion, to avoid excluding a relevant paper, papers that were included by at least 1 researcher were included in the database. Consequently, the following label numbers 1 (included/included), 2 (included/excluded), and 4 (included/not shown) were included in the database. To exclude a paper from the database, we required at least 2 researchers to exclude the paper or that ASReview did not show the paper twice owing to its irrelevance. Therefore, papers with 3 (excluded/excluded) or 6 (not shown/not shown) were excluded from the database.

Papers that were only excluded by 1 researcher, label 5 (excluded/not shown), were uploaded into ASReview and screened a second time by a researcher. All papers that were excluded again (label B) were then excluded from the database, and those that were included were added to the database (label A). All the papers not shown to the researcher in this second screening were excluded owing to their likely irrelevance (label C). The outputs from these screening steps were exported from ASReview, and the data were merged into 1 Microsoft Excel document.

### Quality Assurance of the Database

In this study, false positives (inclusion of an irrelevant paper) were less of a concern, as they could not endanger the integrity of the database, so our strategy prioritized reducing the number of false negatives (exclusion of a relevant paper). To achieve high accuracy with a low rate of false negatives, we implemented a strategy to guarantee the quality and accuracy of the database.

#### Checking for False Negatives

To assess the possibility of false negatives, we conducted an evaluation using 80 papers provided by Health CASCADE Marie Skłodowska-Curie Innovative Training Network, which met our inclusion criteria but were not included in the original data set used to train ASReview. We searched for these papers in our database and noted any paper that was not found as error. We then traced the sources of each paper back through each step of the methodology to determine at which point it was excluded.

Four possibilities of false negatives were considered: double exclusion by 2 researchers, exclusion by 1 researcher and ASReview did not show it to the second researcher, ASReview did not show it to any researcher, or a data management error. In addition, there was a possibility that a paper was missing from the database because it was not originally present in the result of the search, which was not considered a false negative. Any of these situations would have resulted in the absence of a relevant item in the database.

To calculate the false-negative rate, we summed the number of papers from the 4 possible sources of false negatives and divided it by the number of test papers minus the number of test papers that were absent from the original search.

#### Checking for False Positives

To evaluate the potential number of papers in the database that did not adhere to the screening selection criteria (false positives), 3 researchers double screened a random sample of 5% (688/13,760) of the final database. The aim was to identify any papers that should have been excluded. The estimation of the false-positive rate for the full database was calculated by determining the false-positive rate of the screened sample, which is the number of papers excluded divided by the total number of papers screened.

### Analysis of the Database

To gain a better sense of the composition of the final database, we conducted a bibliometric analysis of the included literature. We used Rayyan (Qatar Computing Research Institute), a systematic review manager [28], and VOSviewer (Centre for Science and Technology Studies), a tool for constructing and visualizing bibliometric networks [29], to analyze the frequency of terms in titles and abstracts as well as their co-occurrence. We also used VOSviewer to analyze coauthorship links and authors’ citations to explore how co-creation research is distributed and shared over time. We performed an analysis of the source landscape to identify the dominant fields represented in the database. This involved mapping the source journals and examining their respective disciplines. The full method for replicating this analysis in Rayyan and VOSviewer is presented in Multimedia Appendix 2.

## Results

### Study Selection

**Figure 3:**
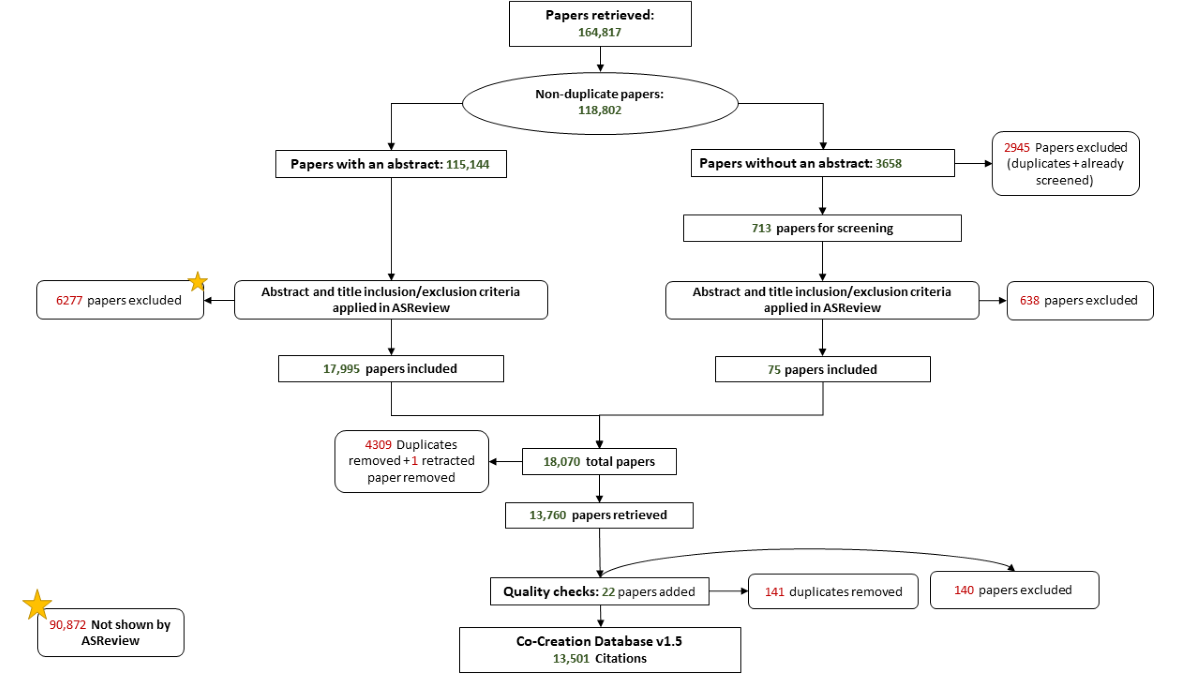
An adapted PRISMA (Preferred Reporting Items for Systematic Reviews and Meta-Analyses) flowchart of the full methodology. The start indicates the citations that were not shown by ASReview to the researchers.

The adapted PRISMA flowchart is shown in Figure 3, and a filled-in PRISMA checklist is provided in Multimedia Appendix 3. On the basis of the initial search and after duplicates were removed, 118,802 papers were identified. As shown in Figure 3, there were 2 parallel screening processes for this literature: one for screening papers with titles and abstracts in ASReview (n=115,144) and another for manual screening of papers without an abstract (n=3658). During the screening assisted by ASReview (left side of Figure 3), a total of 6277 papers were excluded. During the screening of papers without abstracts (right side of Figure 3), these papers were compared manually to check for duplication or presence within the set of papers included or excluded during the ASReview screening, excluding 2945 papers. Then, the nonduplicate papers were screened manually, excluding an additional 638 papers.

After these screening stages, 6915 papers were excluded by the researchers, and 90,872 papers were excluded by ASReview based on irrelevancy. During our data cleaning stage, 4309 duplicates were removed and 1 publication was retracted, resulting in a total of 13,760 papers included in the database. Finally, following the quality check, 22 additional papers were included, 140 more papers were excluded, and 141 duplicates were removed. These steps resulted in a final database that contained 13,501 papers.

### Database Quality

We evaluated the accuracy of the methodology by searching for 80 papers that adhered to our selection criteria but were not included in the original data set used to train ASReview. Of the 80 papers, 58 (72%) papers were found in the database and 22 (28%) were not included in the database. Further analysis of the 22 missing papers revealed that ASReview did not show 6 papers to any researcher during the AI-assisted selection process, whereas 16 papers were not present in the original search results. As a result, the false-negative rate was determined to be 9% (6/64), indicating that 91% (58/64) of the relevant literature was captured by the methodology.

The false-positive rate for the entire database was calculated by determining the false-positive rate of the screened sample, which is the number of papers excluded (n=140) divided by the total number of papers screened (n=688), resulting in a false-positive rate of 20.3%.

### Terminology

The most frequent terms in the title or abstract of a paper in the database were participatory (n=8101) and different variations of the word co-creation (eg, c-ocreation, co-create, co-creating, and co-creators; n=2896). The other terms that appeared in the database were co-production (n=856), user involvement (n=779), co-design (n=760), and public participation (n=463). Finally, the least frequent terms were citizen science (n=184), collaborative design (n=113), public and patient involvement (n=64), and experience-based design (n=11). Co-conception was not present in the database (n=0).

**Figure 4:**
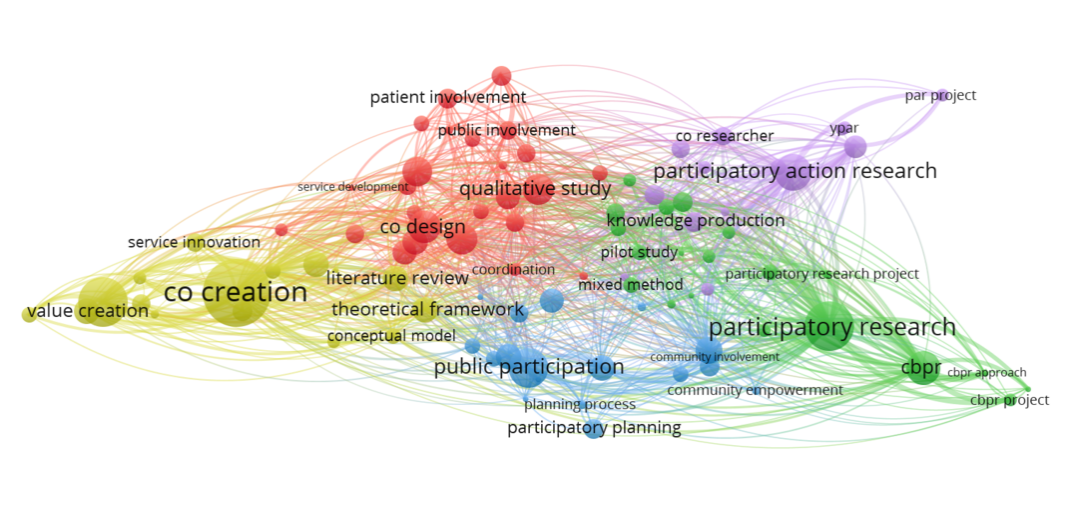
Keyword co-occurrence across the title and abstracts of the papers from January 2010 to November 2022. Each link between 2 keywords represents a co-occurrence. The size of the keyword bubble represents its importance in the number of co-occurrences. Five colors represent 5 clusters. cbpr: community-based participatory research.

The VOSviewer co-occurrence analysis of the keywords in the title and abstracts of the papers in the database is visualized as a network map in Figure 4. This network map included 93 keywords linked to each other 2818 times. VOSviewer also grouped the keywords into 5 clusters [29]. Cluster 1 (in red) contained all forms of co-design, co-production, patient engagement, participatory design, and public involvement. Cluster 2 (in green) contained different forms of community-based participatory research (CBPR), participatory research, and qualitative and quantitative research. Cluster 3 (in blue) contained forms of community, public, and stakeholder development; engagement; participation; and citizen science and citizen participation. Cluster 4 (in yellow) contained forms of co-creation, innovation, and value creation. Cluster 5 (in purple) contained forms of participatory research, participatory action research, and collaborative research. In addition, among these clusters, the main connecting terms were the different forms of engagement, such as user involvement; public or patient involvement; and the less predominant methodologies, such as co-design and co-production. Finally, there were a few methodological terms that linked the clusters, such as the use of theoretical frameworks and models, process planning, mixed methods, collective action, and consultation. A summary of this analysis is provided in Multimedia Appendix 4.

**Figure 5:**
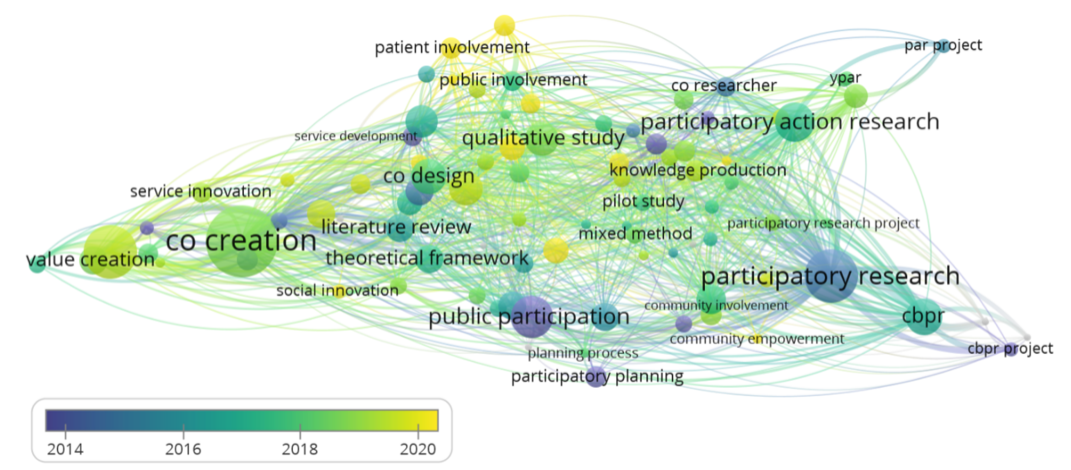
Keyword co-occurrence across the title and abstracts of the papers in the database from January 2010 to November 2022. Each link between 2 keywords represents a co-occurrence. The size of the keyword bubble represents its importance in the number of co-occurrences. The color of the link represents the period where it occurs, and the color of the bubble represents the average of the co-occurrence’s periods. (date range on the displayed scale is automatically adapted by the software based on relevancy). cbpr: community-based participatory research.

The VOSviewer co-occurrence analysis of the keywords in the title and abstracts of the papers in the database is visualized as a network map in Figure 5. Automatically adapted by the software for optimal relevancy [29], the date ranges from 2010 to 2022. The clusters that contained the most recent publications, ranging from 2018 to 2022, were clusters 1, 4, and 5. The most frequent terms in these clusters were co-design, co-production, public involvement, co-creation, innovation, participatory action research, and collaborative research. The older clusters, clusters 2 and 3, ranged from as far back as 2011, and the most recent publication occurred around 2017. Clusters 2 and 3 contained the different forms of participatory research; CBPR; qualitative and quantitative research; community, public, and stakeholder development; engagement; participation; and citizen science. A summary of this analysis is provided in Multimedia Appendix 4.

### Bibliometric Analysis

**Figure 6:**
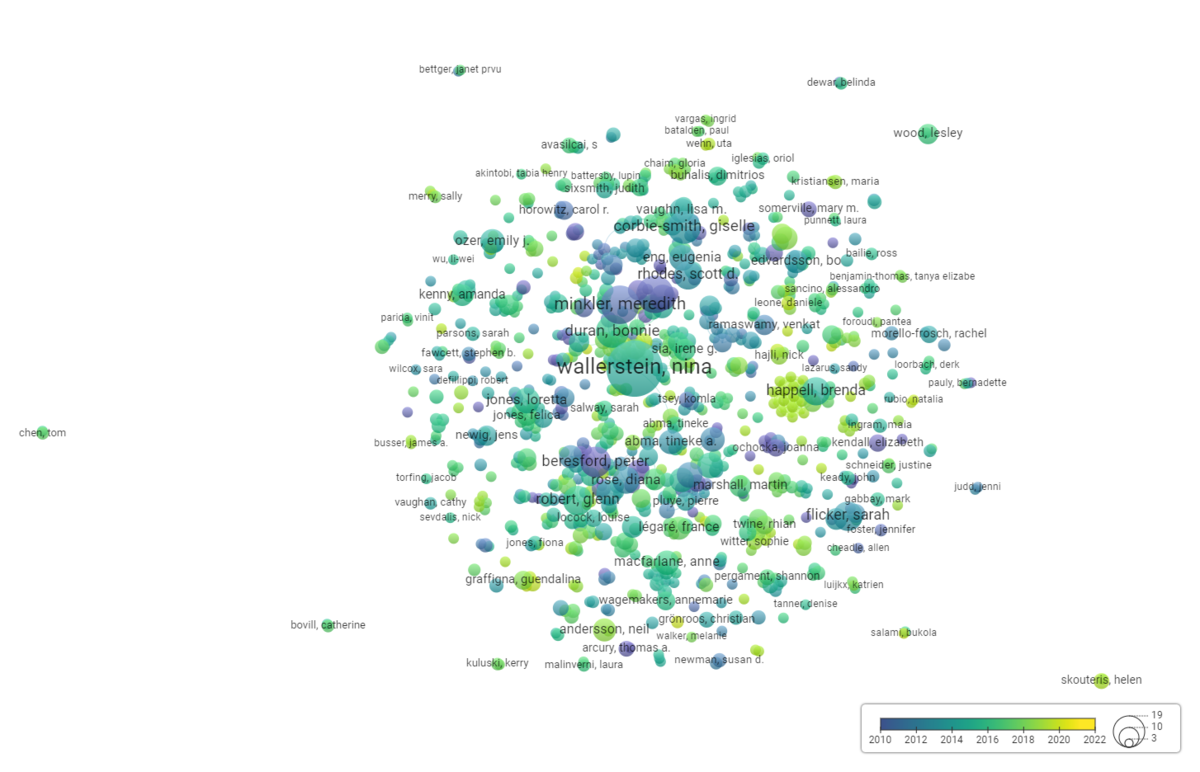
Mapping and clustering of coauthorship of the papers in the database from January 2010 to November 2022. The size of the author’s bubble represents its importance in the number of coauthorships. The color represents the average period where the coauthorships occur.

The VOSviewer analysis of coauthorship is visualized as a network map in Figure 6. The date range was optimized for relevancy by the software [29], spanning from 2010 to 2022. In this network map, there were 1544 links between authors, with an overall total link strength of 3740. We aimed to determine the extent of coauthorship of each author by assessing their total link strength, which represents the number of publications that the author has coauthored. Among the authors, there were 15 authors with a total link strength of 64; hence, we considered all authors with a total link strength of >64 as having the most coauthorship. The 4 authors with the most coauthorship (highest total link strength) were Nina Wallerstein (134/3740, 3.58%), Bonnie Duran (80/3740, 2.14%), Brenda Happell (79/3470, 2.11%), and Julia Bocking (74/3470, 1.98%).

This network map included 18 clusters of 819 authors, with only 2 clusters forming around the 4 authors with the most coauthorships. One cluster included Brenda Happell (a nursing and midwifery researcher) and Julia Bocking (a nursing researcher), with publications from 2015 to 2020. The other cluster included Nina Wallerstein (a community-based participatory and participatory health researcher) and Bonnie Duran (a community-based participatory researcher), with publications from 2011 to 2020. A summary of this analysis is provided in Table 1 and Multimedia Appendix 4.

**Figure 7:**
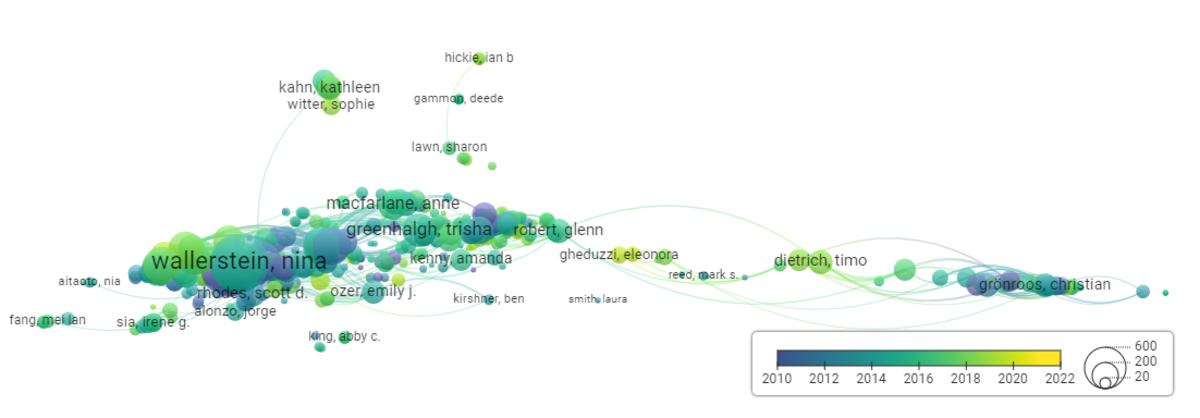
Mapping and clustering of author citations from January 2010 to November 2022. The author’s bubble’s size represents its importance in the number of citations. Each link between 2 authors represents a citation. The color of the link represents the period where the citation occur, and the bubble 1 represents the average periods of the author’s citations.

The VOSviewer analysis of authorship citations is visualized as a network map in Figure 7. Automatically adapted by the software for optimal relevancy [29], the date ranges from 2010 to 2022. This network map included 14 clusters of 389 authors, with a combined total of 121,037 citations among the authors. In this analysis, we were interested in the total citations of each author. The authors with <2500 citations were not included in the list of most-cited authors. On the basis of this criterion, there were 8 authors with the most citations, including Christian Grönroos (3550/121,037, 2.93%), Nina Wallerstein (3083/121,037, 2.55%), Mark S Reed (3045/121,037, 2.52%), Pennie Frow (2955/121,037, 2.44%), Bonnie Duran (2840/121,037, 2.35%), Kaj Storbacka (2792/121,037, 2.31%), Paul P Maglio (2652/121,037, 2.19%), and Meredith Minkler (2538/121,037, 2.09%). These most-cited authors were only grouped into 3 clusters. The first cluster was formed around Christian Grönroos (marketing and economics researcher), Pennie Frow (marketing researcher), Kaj Storbacka (economics researcher), and Paul P Maglio (management researcher). Mark S Reed (environmental governance researcher) was also present in that cluster, but he was on the periphery of it, away from the other authors. The second cluster included Nina Wallerstein and Bonnie Duran, who were both community-based participatory researchers. The third cluster only included Meredith Minkler, a participatory and public health researcher. Meredith’s cluster was very close to Nina’s and Bonnie’s cluster, whereas Christian’s cluster was on the other end of the network map. A summary of this analysis is provided in Table 2 and Multimedia Appendix 4.

**Figure 8:**
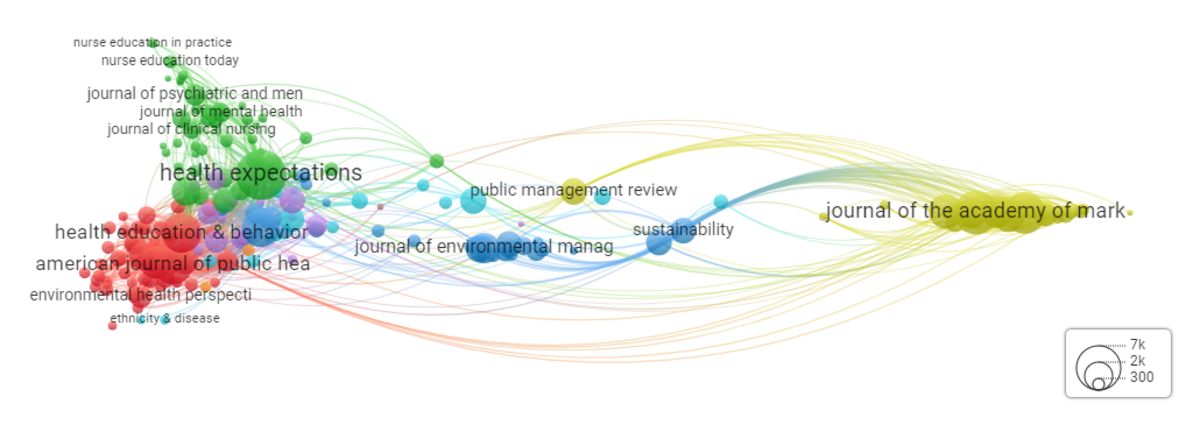
Source landscape mapping from January 1970 to November 2022. Each link between 2 journals represents a citation between 2 journals’ documents. The journal bubble size represents its importance in the number of citations. On 8 clusters in the analysis, 5 clusters are visible through 5 colors.

The VOSviewer analysis of the source journals, called source landscape, is visualized as a network map in Figure 8. This network map included 8 clusters of 188 journals, with a total of 5896 documents. In this analysis, we were interested in the total number of documents per journal, as it indicated the prevailing journals in the database. The journals with >100 documents were considered the predominant journals. On the basis of this criterion, there were 7 top journals, including *Health Expectations* (354/5896, 6%); *Progress in Community Health Partnerships: Research, Education, and Action* (317/5896, 5.38%); the *International Journal of Integrated Care* (167/5896, 2.83%); *Sustainability* (160/5896, 2.71%); *Health Promotion Practice* (149/5896, 2.53%); the *International Journal of Environmental Research and Public Health* (139/5896, 2.36%); *BMJ Open* (119/5896, 2.02%); and *BMC Health Services Research* (113/5896, 1.92%). These predominate journals were grouped into 4 clusters. The first cluster (in green) included *Health Expectations* and *BMJ Open* as well as other less dominant journals about different forms of health research. The second cluster (in red) included *Progress in Community Health Partnerships: Research, Education, and Action*; *Health Promotion Practice*; and the *International Journal of Environmental Research and Public Health*. This cluster also included different public health journals. The third cluster (in purple) included the *International Journal of Integrated Care* and *BMC Health Services Research* as well as less dominant journals about health services, research, and policy. The fourth cluster (in blue) included *Sustainability* and other less dominant journals about social and environmental sciences and action research. A summary of this analysis is provided in Table 3 and Multimedia Appendix 4.

**Table 1:** Authors and field of research (based on Figure 6).

Authors	Field	Total link strength	Total links (co-authors), n	Average publication year
Nina Wallerstein	Community-based participatory research and participatory health research	134	50	2015
Bonnie Duran	Community-based participatory research	80	19	2017
Brenda Happell	Nursing and midwifery research	79	19	2015
Julia Bocking	Nursing research	74	18	2019

**Table 2:** Author citations and field of research (based on Figure 7).

Most-cited authors	Field	Citations (n=121,037), n (%)	Average publication year
Christian Grönroos	Marketing and economics research	3550 (2.93)	2015
Nina Wallerstein	Community-based participatory research	3083 (2.55)	2015
Mark S Reed	Environmental governance research	3045 (2.52)	2013
Pennie Frow	Marketing research	2955 (2.44)	2012
Bonnie Duran	Community-based participatory research	2840 (2.35)	2017
Kaj Storbacka	Economics research	2792 (2.31)	2010
Paul P Maglio	Management research	2652 (2.19)	2011
Meredith Minkler	Participatory and public health research	2538 (2.09)	2011

**Table 3:** Characterizing the source landscape analysis in Figure 8.

Journal (source)	Papers (n=5896), n (%)	Citations (n=154,945), n (%)	Average publication year	Cluster number (color)
*Health Expectations*	354 (6)	9894 (6.39)	2017	2 (green)
*Progress in Community Health Partnerships: Research, Education, and Action*	317 (5.38)	3094 (2)	2014	1 (red)
*International Journal of Integrated Care*	167 (2.83)	120 (0.08)	2018	5 (purple)
*Sustainability*	160 (2.71)	1851 (1.19)	2020	3 (blue)
*Health Promotion Practice*	149 (2.53)	3173 (2.05)	2014	1 (red)
*International Journal of Environmental Research and Public Health*	139 (2.36)	1110 (0.72)	2020	1 (red)
*BMJ Open*	119 (2.02)	931 (0.6)	2019	2 (green)
*BMC Health Services Research*	113 (1.92)	1900 (1.23)	2018	5 (purple)

## Discussion

### Overview

This study consolidates multidisciplinary literature on co-creation into a curated and open-access database. This database provides a trustworthy source of information about co-creation, which can save time and resources that would otherwise be spent searching through a vast body of literature. This database can facilitate the identification of research gaps and enable the exploration of transdisciplinary co-creation research beyond what can be learned from a traditional systematic review. Moreover, our source landscape analysis suggests that this database can offer particular benefits for co-creation researchers in the field of health sciences, given the predominance of health-related journals in the database. Finally, our novel AI-assisted methodology based on systematic reviews dealt with the ambiguities and terminological diversity surrounding co-creation, and made it possible to consolidate literature about a fragmented concept from different fields.

### Principal Findings

#### Overview

A notable strength of this study is its incorporation of diverse perspectives from a wide range of researchers as well as its collaboration with ASReview. This study marks the first occasion in which an AI-assisted screening process was used to manage the extensive and multifaceted concept of co-creation [10,30]. The application of AI tools in literature synthesis is a recent development; nevertheless, we successfully integrated it into well-established literature synthesis methodologies, including systematic and Cochrane reviews [19,31].

#### High-Quality Database

Another strength of this study is that we conducted a thorough quality assurance process throughout. This particular focus on checking for false positives and false negatives is not usually completed for systematic reviews, even in the most advanced methodologies such as Cochrane reviews [32]. The quality of the database exceeded our initial expectations. Even the best manual screening that follows stringent procedures contains elements of subjectivity and error. However, our quality assurance process showed that we could achieve high sensitivity and specificity. Our false-negative rate was 9% (6/64), which is the average error rate lower than what is achievable by a human alone, which is about 10% on average [33]. Our false-positive rate was 20.3% (140/688), meaning that approximately 20% of the database contained irrelevant papers. This is the result we aimed for, as our methodology prioritized inclusion over exclusion.

#### Key Terms for Co-Creation

The frequency analysis of the final database demonstrated the presence of commonly used terms in co-creation research such as co-creation, co-design, and co-production as well as user involvement. Furthermore, the VOSviewer co-occurrence analysis of keywords revealed the prominence of diverse forms of participatory research, including, but not limited to, participatory action research, CBPR, and other forms of participatory research. This reveals that our unified concept of co-creation includes all the coapproaches (co-creation, co-design, and co-production) as well as participatory research and user involvement. Therefore, it is important to not exclude these research methodologies when investigating co-creation.

#### Knowledge Fragmentation

Our bibliometric analysis indicated that researchers in the field of co-creation predominantly cite papers from the fields of management, environmental sciences, CBPR, marketing, and economics. The source landscape analysis revealed that most journals within our database were health research focused, including health services research, health promotion, and integrated care. In addition, the coauthorship clustering suggested limited collaboration across fields, with authors tending to group by their field of research. This observation may be attributed to the vast volume of literature on co-creation, which could make it challenging for researchers to thoroughly investigate all relevant literature. As a result, researchers may focus primarily on papers from their field, thus missing opportunities for cross-fertilization between fields.

In addition, we observed a homogeneous network for the co-occurrence of keywords within the database, indicating researchers are using many, but similar, terms. This phenomenon may be because co-creation is a broad and diffuse concept that can be approached from various angles and contexts, and thus, researchers may be using different terms to emphasize different aspects of the concept. Although this could appear as a positive indicator for multidisciplinary approaches, this similarity in terminology may also contribute to the potential knowledge fragmentation. Specifically, the interchangeable use of terms may result in missed opportunities for researchers to engage with literature outside of their field, as they may not be aware of alternative terms being used to describe similar concepts.

These analyses further suggest the potential knowledge fragmentation about co-creation owing to the diverse and interchangeable use of terminology, researchers working within silos, and the low cooperation and poor communication between researchers. Therefore, our findings suggest the need for greater attention to be paid to standardizing and clarifying the terminology used in co-creation research to prevent the further fragmentation of the co-creation concept.

#### Open-Access Database

As our methodology was successful, another key result is the publication of an openly accessible and downloadable database, which is available for individuals or organizations to use [34]. This study provides methodological rigor and validity for the database, so users can conduct their research within the database with confidence. In addition, by making this database open access, researchers and practitioners can test its usability and invite additional experts to participate in the process of validating and expanding its content.

### Comparison With Prior Work

Vargas et al [9] argued that co-creation is a method of participatory action research, and co-creation includes co-production and co-design as submethods. On the basis of the keyword co-occurrence analyses in this study, we can infer that indeed participatory research is a key component of co-creation, whereas co-design and co-production may serve as submethods. These findings, and the literature contained in the database, suggest that co-creation is predominantly composed of the coapproaches and various forms of participatory research.

This study highlights the need for clarification of the conceptual differences among methodologies for collaborative creative problem-solving among diverse stakeholders. Masterson et al [10] recently emphasized the need to focus on underlying principles and values when seeking to coproduce and co-design. Our study adopted principles of co-creation, such as collective creativity, a broad range of relevant stakeholders, creative problem-solving, and desired outcomes, to form a comprehensive definition of co-creation that can unify different terminologies. Our keyword analysis revealed a high degree of co-occurrence between terms, indicating a shared understanding of co-creation across the database. Therefore, future analysis of this database can help to uncover key values and principles of co-creation, which can begin to address the fragmentation of knowledge about co-creation.

Finally, the recent popularity of the terminology co-creation is also visualized in our database, as it occurs most frequently in the latest publications, with value co-creation emerging around 2017. In contrast, various forms of participatory research have been present since 2014, with public participation appearing as early as 2010. These findings may provide support for Bauman’s [7] assertion that co-creation is a fashionable term that can be traced back to CBPR as well as the argument that co-creation has its roots in participatory action research [9]. These findings highlight the importance of further research to explore co-creation and its relationship with participatory research methodologies. Such research can help to develop a more nuanced understanding of the different approaches to co-creation and support the development of effective strategies to facilitate its application and knowledge exchange.

### Limitations

Although we achieved our objectives and created a high-quality database, our approach has some limitations including missing metadata, the potential overinclusion of papers, and the fact that the database only represents a snapshot in time. Furthermore, with the absence of user-friendly application programming interfaces, it was necessary to manually download the vast number of search results. Manually processing this vast amount of data increased the risk of making mistakes. In addition, owing to the anticipated volume of literature, we limited ourselves to only searching CINAHL, PubMed, and ProQuest, and therefore, some appropriate literature may have been missed. In addition, to ensure the rigor and quality of our study, we made a deliberate decision to include only published and peer-reviewed literature in our search strategy. However, we recognize that this approach may have resulted in limitations in the amount and type of knowledge included in our final database, as we did not perform a gray literature search.

### Next Steps

#### Future Iterations and Usability

This study successfully created a high-quality database, and to maintain its relevancy, it is necessary to incorporate additional literature from other sources. However, it is important to note that the novel AI-assisted methodology used in this study can be time-consuming and resource intensive, making it challenging to repeat the process annually. To expedite the process of updating the database, an approach is to use a classification model to perform the selection process by identifying relevant papers from a new set of literature. This approach can only be implemented once the model is trained on the existing high-quality co-creation database. As a test, an initial update has been conducted that incorporates literature from Scopus and Web of Science. Such an approach holds promise for ensuring that the database remains up to date with relevant literature and could facilitate more efficient and effective database maintenance in the future.

However, the assessment of the accuracy of the selection process is lower than the novel AI-assisted methodology applied in this study. Therefore, we plan to call on the scientific community to help us identify irrelevant papers within the database and any relevant papers missing from the database. This process can improve our classification model for future updates. The details about this test and the classification model are provided in Multimedia Appendix 5.

#### Concept and Theoretical Analyses

Our study aimed to provide a comprehensive overview of the existing literature on co-creation, which serves as a foundational step for future research in this area. Although we have presented the scope and nature of the literature, further analysis is needed to fully understand the concepts and themes present in the database. In this regard, future research can benefit from conducting a more in-depth concept and theoretical analysis of the literature in our database, which could contribute to the advancement of co-creation research.

### Conclusions

This study produced a high-quality curated open-access database consolidating a vast amount of literature about co-creation through the design and implementation of a novel AI-assisted methodology based on systematic reviews. In doing so, the study demonstrated that it is possible to consolidate knowledge about diffuse concepts using human-AI collaboration.

The open-access database is a curated snapshot of literature from 1970 to 2022, with false-negative rates similar to that of manually screened literature review. It also made the amount of information manageable for performing searches, while still containing a diversity of literature, as shown in our analysis. This allows the possibility for cross-fertilization and learning from studies where co-creation was applied in different fields and different geographic locations. Also, through the bibliometric analysis, this study also generates clarity about the current co-creation landscape. We encourage researchers to explore and use this database and reference appropriate papers when researching co-creation.

## Appendix

Multimedia Appendix 1 Search strategy and results.

Multimedia Appendix 2 Rayyan and VOSviewer analysis.

Multimedia Appendix 3 PRISMA (Preferred Reporting Items for Systematic Reviews and Meta-Analyses) 2020 checklist.

Multimedia Appendix 4 VOSviewer results.

Multimedia Appendix 5 Updating the database.

## Abbreviations

AI: artificial intelligence
CBPR: community-based participatory research
PRISMA: Preferred Reporting Items for Systematic Reviews and Meta-Analyses

## Data Availability

We have made both generated databases, version 1.5 [35] and version 2.0 [36], freely available under the name *Co-Creation Database* within the Health CASCADE’s Zenodo repository.
