# Supplementary material for: Establishing a Health CASCADE–Curated Open-Access Database to Consolidate Knowledge About Co-Creation: Novel Artificial Intelligence–Assisted Methodology Based on Systematic Reviews
Source: J Med Internet Res. 2023 Jul 18;25:e45059. doi: 10.2196/45059 (PMC10394503; doi:10.2196/45059)
Supplement: Multimedia Appendix 1 [file jmir_v25i1e45059_app1.docx]

**Multimedia Appendix 1. Search Strategy and Results**

Search for all relevant data with the following search strategy, which was adopted and tailored to each database. The results from each database can be found below.

Keywords (in English):

1. Co-creat* (TARGETS: co-creation, co-create, co-creating, co-creators, Mode 2 co-creation, agile co-creation, value co-creation)
2. Co-conception
3. Co-production
4. “Public and patient involvement”
5. “Public participation”
6. Participatory
7. “Experience based design”
8. Co-design
9. “User involvement”
10. “Collaborative design”
11. “Citizen science”

Search limits:

1. Excluded the following type of materials: Blogs, Websites, Podcasts, Biography, Conference abstracts, papers and proceedings, dissertations and theses, letter(s) to editor, newspapers, magazines, pamphlets or brochures, speeches, lectures, or presentations, working papers, audio and video works, artistic and aesthetic works, Encyclopaedia and reference works, or essays and interviews.
2. Only include materials that are written in English.
3. Only include materials published on or after January 1, 1970 (Koning et al., 2016).

Tailored Searches per Database:

**1) CINAHL:** We have full access to this database. This database includes a mesh-building advanced search methodology and is not included in ProQuest. CINAHL covers nursing, biomedicine, health sciences, alternative/complementary medicine, occupational therapy, physical therapy and allied health, biomedicine, and healthcare.

- **Databases:** AMED – The Allied and Complementary Medicine Database; British Education Index; Child Development & Adolescent Studies; eBook Collection (EBSCOhost); Education Abstracts (H.W.Wilson); Educational Administration Abstracts; ERIC; GreenFILE; Health Source: Nursing/Academic Edition; Library, Information Science & Technology Abstracts; MEDLINE; Regional Business News; CINAHL Plus with Full Text; Business Source Premier; and SPORTDiscus with Full Text.
- **Removed keyword:** co-conception as there were no results in CINAHL
- We did not use MeSH terms because we wanted to make it more comparable to the other two databases (ProQuest and PubMed).
- **Search Terms:** co-creat* OR “citizen science” OR co-production OR ("public and patient involvement") OR “public participation” OR participatory OR “Experience based design” OR co-design OR “user involvement” OR “collaborative design”
- **Limiters:** Published Date: 19700101-20221131; Peer Reviewed; References Available; Document Type: Editorial, Journal Article, Meeting-Paper, Meeting-Report, Report, Review; Language: English; Publication Type: Academic Journal, Book; Document Type: Book Review, Case Study, Comparative Treatment, Editorial, Journal Article, Literature Reviews, Research Reports, Research Reviews; Language: English; Publication Type: academic journal, book, case study, government document, health report, report, review; Document Type: article, book, case study, report, science experiment; Language: English; Publication Type: Academic Journal, Book, Review; Document Type: Article, Book Review, Case, Case Study, Editorial, Excerpt from Book; Publication Type: Academic Journal, Health Report; Publication Type: Books, Collected Works (All), Journal Articles, Reports (All); Language: English; Publication Type: Academic Journal, Book/Monograph; Publication Type: Academic Journal, Review
- **Search Modes:** Find all my search terms
- **Results (**as of 01.12.2021**):** 12,313 papers

**2) Pubmed:** We have full access to this database. This database uses a mesh-building advanced search methodology and is not included in ProQuest. This database focuses on biomedical literature from Medline, life science journals, and online books.

- **Removed keyword:** co-conception as there were no results in PubMed
- **Excluded materials:** Autobiography, Biography, Comment, Lecture, Letter, News newspaper article, Personal narrative, Portrait, Preprint, Retracted publications, Webcast, Interview, Electronic supplementary materials, Directory, Conference or congress, Comment, Address, Corrected and republished articles, and Published erratum.
- **Full Search Field:** ("co creat*"[Title/Abstract] OR "co-production"[Title/Abstract] OR "public and patient involvement"[Title/Abstract] OR "public participation"[Title/Abstract] OR "Participatory"[Title/Abstract] OR "experience based design"[Title/Abstract] OR "co-design"[Title/Abstract] OR "user involvement"[Title/Abstract] OR "collaborative design"[Title/Abstract] OR "citizen science"[Title/Abstract]) AND ((booksdocs[Filter] OR casereports[Filter] OR classicalarticle[Filter] OR clinicalstudy[Filter] OR clinicaltrial[Filter] OR clinicaltrialprotocol[Filter] OR clinicaltrialphasei[Filter] OR clinicaltrialphaseii[Filter] OR clinicaltrialphaseiii[Filter] OR clinicaltrialphaseiv[Filter] OR veterinaryclinicaltrial[Filter] OR comparativestudy[Filter] OR correctedandrepublishedarticle[Filter] OR dataset[Filter] OR editorial[Filter] OR electronicsupplementarymaterials[Filter] OR evaluationstudy[Filter] OR festschrift[Filter] OR governmentpublication[Filter] OR guideline[Filter] OR historicalarticle[Filter] OR introductoryjournalarticle[Filter] OR journalarticle[Filter] OR meta-analysis[Filter] OR observationalstudy[Filter] OR veterinaryobservationalstudy[Filter] OR overall[Filter] OR practiceguideline[Filter] OR pragmaticclinicaltrial[Filter] OR randomizedcontrolledtrial[Filter] OR researchsupportamericanrecoveryandreinvestmentact[Filter] OR researchsupportnihextramural[Filter] OR researchsupportnihintramural[Filter] OR researchsupportnonusgovt[Filter] OR researchsupportusgovtnonphs[Filter] OR researchsupportusgovtphs[Filter] OR researchsupportusgovernment[Filter] OR review[Filter] OR scientificintegrityreview[Filter] OR systematicreview[Filter] OR technicalreport[Filter] OR twinstudy[Filter] OR validationstudy[Filter]) AND (humans[Filter] OR animal[Filter]) AND (1970/1/1:3000/12/12[pdat]) AND (english[Filter]))
- **Results (**as of 01.12.2021**):** 15,705 papers

**3) ProQuest:** We have access to the full list of databases accessible via ProQuest. Additionally, ProQuest has a user-friendly advanced search method and includes multidisciplinary databases that span academia, corporate, government, public, and schools around the world

- **Included document type:** Evidence based healthcare, literature review, book, statistics/data report, instructional material/guideline, reference document, book chapter, industry report, and technical report.
- Language: English
- **Source type:** Scholarly Journals, Reports, Books, Other sources, and trade journals.
- **Limit to:** Peer reviewed
- **Databases searched (17):** APA PsycArticles, APA PsycInfo, Art, Design & Architecture Collection, British Periodicals, Coronavirus Research Database, Early Modern Books, Ebook Central, Entertainment Industry Magazine Archive, Humanities Index, Periodicals Archive Online, ProQuest One AcademicTrial - Limited time only, PTSDpubs, SciTech Premium Collection, Social Science Premium Collection, Sports Medicine & Education Index, The Vogue Archive, and The Women's Wear Daily Archive
- **Full Search Field:** (noft(co-creat*) OR noft(co-conception) OR noft(co-production) OR noft("public and patient involvement") OR noft("public participation") OR noft(participatory) OR noft("experience based design") OR noft(co-design) OR noft("user involvement") OR noft("collaborative design") OR noft("citizen science")) AND (at.exact(("Article" OR "Feature" OR "Report" OR "Undefined" OR "Book" OR "Review" OR "Book Chapter" OR "Editorial" OR "General Information" OR "Case Study" OR "Reference Document" OR "Evidence Based Healthcare" OR "Literature Review" OR "Instructional Material/Guideline" OR "Government & Official Document" OR "Statistics/Data Report" OR "Letter to the Editor" OR "Industry Report" OR "Technical Report") NOT ("News" OR "Commentary" OR "Conference" OR "Conference Proceeding" OR "Speech/Lecture" OR "Correspondence" OR "Interview" OR "Correction/Retraction" OR "Front Page/Cover Story" OR "Bibliography" OR "Transcript" OR "Company Profile" OR "Dissertation/Thesis" OR "Working Paper/Pre-Print" OR "Obituary" OR "Biography" OR "Business Case" OR "Website/Webcast" OR "Financial Materials" OR "Conference Paper" OR "Front Matter" OR "Memoir/Personal Document" OR "Directory" OR "Market Research" OR "Blog" OR "Credit/Acknowledgement" OR "Fiction" OR "Poem" OR "Image/Photograph" OR "Prose" OR "Translation" OR "Back Matter" OR "Essay" OR "Fund/Grant/Fellowship/Award" OR "Editorial Cartoon/Comic" OR "Illustration" OR "Pamphlet/Ephemera" OR "Play" OR "Recipe" OR "Standard" OR "Table Of Contents" OR "Table of Contents")) AND stype.exact(("Scholarly Journals" OR "Wire Feeds" OR "Trade Journals" OR "Books" OR "Reports" OR "Other Sources" OR "Government & Official Publications") NOT ("Newspapers" OR "Dissertations & Theses" OR "Conference Papers & Proceedings" OR "Magazines" OR "Working Papers" OR "Blogs, Podcasts, & Websites" OR "Encyclopedias & Reference Works" OR "Speeches & Presentations" OR "Audio & Video Works")) AND la.exact("ENG") AND pd(19700101-20221231) AND PEER(yes))
- **Results (**as of December 2 2021**):** 141,215 papers - the search results were downloaded until December 9, 2021.
