# Supplementary material for: Establishing a Health CASCADE–Curated Open-Access Database to Consolidate Knowledge About Co-Creation: Novel Artificial Intelligence–Assisted Methodology Based on Systematic Reviews
Source: J Med Internet Res. 2023 Jul 18;25:e45059. doi: 10.2196/45059 (PMC10394503; doi:10.2196/45059)
Supplement: Multimedia Appendix 2 [file jmir_v25i1e45059_app2.docx]

**Multimedia Appendix 2. Rayyan and VOSviewer Analysis**

**Keyword analysis in Rayyan**

The full database was uploaded into an online and free tool called Rayyan. Once uploaded, all the keywords were input into the ‘keyword for include’ feature of Rayyan. This provided the number of citations that this term was present in the title or abstract. The final dataset was exported into CSV format and was analyzed to create a grouping around the key terms: co-creat*, co-production, co-design, citizen science, collaborative design, experience based design, participatory, public and patient involvement, and public participation. The final numbers were calculated as frequency and displayed in a bar graph.

**VOSviewer Analyses**

Analysis of the co-occurrence of terms in the title and abstract:

1. Uploaded v1.5 RIS file into VOSviewer
2. Excluded abstract markers and copyright markers
3. Counting method: selected binary counting, because it matters whether the term was in the paper or not, not about how often it was mentioned in that paper (frequency)
   1. Binary counting = only the presence or absence of the term in the document matters. The number of occurrences of the term in a document is not taken into account.
4. Minimum number of occurrences of the term: 64 (based on the outcomes of the Rayyan assessment, the most prominent keywords have been mentioned a minimum of 64 times)
5. Number of terms to be selected: 640
6. Terms included if they relate to any form of methodology:
   - Value co creation process
   - Value creation
   - Open innovation
   - Co creator
   - Service innovation
   - Innovation process
   - Design methodology approach
   - Cbpr priniciple
   - Co creation
   - Cbpr approach
   - Co creation process
   - Cbpr project
   - Ypar
   - Youth participatory action research
   - Empirical research
   - Cbpr
   - Social innovation
   - Cocreation
   - Par project
   - Citizen participation
   - Participatory research approach
   - Public participation
   - Participatory research
   - Needs assessment
   - Ppi
   - Service design
   - Scoping review
   - Stakeholder participation
   - Participatory research project
   - Study protocol
   - Intervention development
   - Service user involvement
   - Recruitment
   - Participatory research method
   - Process evaluation
   - Systematic review
   - Policy making
   - Patient participation
   - Patient involvement
   - Pilot study
   - Public engagement
   - Public involvement
   - Systematic literature review
   - Conceptual model
   - Patient engagement
   - Decision making process
   - Participatory action research project
   - Service development
   - Par
   - Community engagement
   - User participation
   - Community empowerment
   - Participatory planning
   - Mixed method
   - User involvement
   - Community involvement
   - Social science
   - Knowledge production
   - Co production
   - Co researcher
   - Design process
   - Citizen science
   - Literature review
   - Participatory action research
   - Participatory action research approach
   - Stakeholder involvement
   - Community participation
   - Theoretical framework
   - Quantitative data
   - Qualitative interview
   - Planning process
   - Participatory design
   - Community development
   - Collective action
   - Co design process
   - Qualitative data
   - Qualitative study
   - Collaborative research
   - Knowledge translation
   - Coproduction
   - Critical reflection
   - Stakeholder engagement
   - Action research
   - Participatory process
   - Consultation
   - Co design
   - Participatory methodology
   - Coordination
   - Iterative process
   - Collaborative

Then we clicked ‘finished’ to generate the figure.

To visualize the results over time, the ‘Color’ parameter was changed to ‘avg.pub.year’ (2010 – 2022)

Analysis of the Co-authorship in VOSviewer

Since our methodology to create the database only used the abstract, titles, authors, and DOI, most of the other bibliographic metadata was missing. However, 84% of the papers have a DOI, so we used the option to do a cross-referencing (Crossref’s API) directly via the VOSviewer, and retrieved the metadata of 11,243 papers out of the 13,501 papers.

The co-authorships links mapping is built around the number of publications two researchers co-authored. For the analysis, we included only authors with at least four documents to reduce the links to generate a legible map.

Then we set the following parameters to generate the map:

- Normalization method = Association strength
- Minimum cluster size = 1
- Merge small clusters
- Size = Documents
- Size Variation = 0.5
- Color = avg.pub.year (2010 – 2022)
- Maximum label length = 30
- Minimum strength = 0
- Maximum links = 1000
- Curved links
- Cluster colors = Viridis

In this analysis, a link represents the number of publications researchers have co-authored. The Total link strength attribute indicates the total strength of the co-authorship links of a given researcher with other researchers.

Analysis of Citations in VOSviewer

Since our methodology to create the database only used the abstract, titles, authors and DOI, most of the other bibliographic metadata was missing. However, 84% of the papers have a DOI, so we used the option to do a cross-referencing (Crossref’s API) directly via the VOSviewer, and retrieved the metadata of 11,243 papers out of the 13,501 papers.

This analysis was built around the number of times authors cited each other. For the analysis, we included only authors with at least five documents to reduce the links and generate a legible map.

Then we set the following parameters to generate the map:

- (Visualization) Scale = 1
- Normalization method = Association strength
- Minimum cluster size = 1
- Merge small clusters
- Size = Total link strength
- Size Variation = 0.5
- Color = avg.pub.year (2010 – 2022)
- Maximum label length = 30
- Minimum strength = 0
- Maximum links = 1000
- Curved links
- Cluster colors = Viridis

Analysis of the Source Landscape in VOSviewer

Since our methodology to create the database only used the abstract, titles, authors, and DOI, most of the other bibliographic metadata was missing. However, 84% of the papers have a DOI, so we used the option to do a cross-referencing (Crossref’s API) directly via the VOSviewer, and retrieved the metadata of 11,243 papers out of the 13,501 papers.

We performed a *sources landscape* mapping exercise, which was built around the number of times documents from one source cited documents from another source. For the analysis, we included only the sources with at least eleven documents.

Then we set the following parameters to generate the map:

- (Visualization) Scale = 1
- Normalization method = Association strength
- Minimum cluster size = 1
- Merge small clusters
- Size = Citations
- Size Variation = 0.5
- Color = Clusters
- Maximum label length = 30
- Minimum strength = 0
- Maximum links = 1000
- Curved links
