# Supplementary material for: Establishing a Health CASCADE–Curated Open-Access Database to Consolidate Knowledge About Co-Creation: Novel Artificial Intelligence–Assisted Methodology Based on Systematic Reviews
Source: J Med Internet Res. 2023 Jul 18;25:e45059. doi: 10.2196/45059 (PMC10394503; doi:10.2196/45059)
Supplement: Multimedia Appendix 4 [file jmir_v25i1e45059_app4.docx]

**Multimedia Appendix 4. Results of the VOSviewer Analysis**

**Keywords**

Table 1. Characterization of the keyword clusters based on Figures 4 and 5

| **Cluster Number / Color** | **Predominate Term(s)** | **Average Publication Year (2010 – 2021)** | **Number of co-occurrences (links)** |
| --- | --- | --- | --- |
| Cluster 1 / Red | co-production, co-design, user involvement, qualitative study | 2019 | 88 |
| Cluster 2 / Green | participatory research and CBPR | 2015 | 89 |
| Cluster 3 / Blue | public participation, participatory process, community engagement, and citizen participation | 2011 | 67 |
| Cluster 4 / Yellow | co-creation and value co-creation | 2019 | 87 |
| Cluster 5 / Purple | Participatory action research | 2017 | 86 |

**Co-authorship**

Table 2: Coauthorship results based on Figure 6

| **Authors** | **Total link strength  (n = 3740), n (%)** | **Totals links (number of co-authors)** | **Cluster number** | **Average Publication year** |
| --- | --- | --- | --- | --- |
| Nina Wallerstein | 134/3740, 3.58% | 50 | 10 | 2015 |
| Bonnie Duran | 80/3740, 2.14% | 19 | 10 | 2017 |
| Brenda Happell | 79/3470, 2.11% | 19 | 8 | 2015 |
| Julia Bocking | 74/3470, 1.98% | 18 | 8 | 2019 |

**Authors**
*Table 3. Author citations based on Figure 7*

| **Most-cited Authors** | **Citations (n=121,037), n (%)** | **Documents** | **Cluster number** | **Average Publication Year** |
| --- | --- | --- | --- | --- |
| Christian Grönroos | 3550 (2.93) | 7 | 2 | 2015 |
| Nina Wallerstein | 3083 (2.55) | 43 | 10 | 2015 |
| Mark S. Reed | 3045 (2.52) | 6 | 2 | 2013 |
| Pennie Frow | 2955 (2.44) | 5 | 2 | 2012 |
| Bonnie Duran | 2840 (2.35) | 20 | 10 | 2017 |
| Kaj Storbacka | 2792 (2.31) | 5 | 2 | 2010 |
| Paul P. Maglio | 2652 (2.19) | 6 | 2 | 2011 |
| Meredith Minkler | 2538 (2.09) | 25 | 5 | 2011 |

**Source Landscape:**

Table 4. Characterizing the Source Landscape based on Figure 8

| **Source** | **Papers**  **(n=5896), n (%)** | **Citations**  **(n=154,945), n (%)** | **Avg. Publication Year** | **Cluster number/color** |
| --- | --- | --- | --- | --- |
| Health Expectations | 354 (6) | 9894 (6.39) | 2017 | 2 / green |
| Progress in Community Health Partnerships: research, education and action | 317 (5.38) | 3094 (2) | 2014 | 1 / red |
| International Journal of Integrated Care | 167 (2.83) | 120 (0.08) | 2018 | 5 / purple |
| Sustainability | 160 (2.71) | 1851 (1.19) | 2020 | 3 / blue |
| Health Promotion Practice | 149 (2.53) | 3173 (2.05) | 2014 | 1 / red |
| International Journal of Environmental Research and public health | 139 (2.36) | 1110 (0.72) | 2020 | 1 / red |
| BMJ Open | 119 (2.02) | 931 (0.6) | 2019 | 2 / green |
| BMC Health Services Research | 113 (1.92) | 1900 (1.23) | 2018 | 5 / purple |
