# Supplementary material for: Establishing a Health CASCADE–Curated Open-Access Database to Consolidate Knowledge About Co-Creation: Novel Artificial Intelligence–Assisted Methodology Based on Systematic Reviews
Source: J Med Internet Res. 2023 Jul 18;25:e45059. doi: 10.2196/45059 (PMC10394503; doi:10.2196/45059)
Supplement: Multimedia Appendix 5 [file jmir_v25i1e45059_app5.docx]

**Multimedia Appendix 5. Updating the Database**

Version 2.0 of the co-creation database includes references from Scopus and Web of Science from January 1970 to March 2023, with an update of the previous databases used for version 1.5 from December 2021 to March 2023.

To do this update, we use a classification model that we trained with the references obtained from version 1.5. The model decided which references to include.

# 1. Search strategy:

We used the same criteria as the one for version 1.5. You can find all the details below:

## Scopus

**Full Search Field:** ( TITLE-ABS-KEY ( "co-creat*" OR {co-production} OR {public involvement} OR {patient involvement} OR {public participation} OR {participatory} OR {experience based design} OR {co-design} OR {user involvement} OR {collaborative design} OR {citizen science} ) ) AND ( EXCLUDE ( DOCTYPE,"no" ) OR EXCLUDE ( DOCTYPE,"sh" ) OR EXCLUDE ( DOCTYPE,"er" ) OR EXCLUDE ( DOCTYPE,"le" ) OR EXCLUDE ( DOCTYPE,"dp" ) OR EXCLUDE ( DOCTYPE,"tb" ) OR EXCLUDE ( DOCTYPE,"rp" ) OR EXCLUDE ( DOCTYPE,"ab" ) OR EXCLUDE ( DOCTYPE,"Undefined" ) ) AND ( EXCLUDE ( PUBYEAR,1969) OR EXCLUDE ( PUBYEAR,1968) OR EXCLUDE ( PUBYEAR,1967) OR EXCLUDE ( PUBYEAR,1965) OR EXCLUDE ( PUBYEAR,1964) OR EXCLUDE ( PUBYEAR,1960) OR EXCLUDE ( PUBYEAR,1958) OR EXCLUDE ( PUBYEAR,1955) OR EXCLUDE ( PUBYEAR,1954) OR EXCLUDE ( PUBYEAR,1953) OR EXCLUDE ( PUBYEAR,1950) OR EXCLUDE ( PUBYEAR,1949) ) AND ( LIMIT-TO ( LANGUAGE,"English" ) ) AND ( EXCLUDE ( DOCTYPE,"cr" ) OR EXCLUDE ( DOCTYPE,"ed" ) )

Date: 10/04/2023 ; 153,656 document results

## WoS

**Hyperlink:** <https://www.webofscience.com/wos/woscc/summary/bff10c4f-348f-4c1c-a262-1645059d59f0-802ff8d3/relevance/1>

**Full Search Field:** (ALL=("co-creat*" OR "co-production" OR "public participation" OR "public involvement" OR "patient involvement" OR "participatory" OR "experience based design" OR "co-design" OR "user-involvement" OR "collaborative design" OR "citizen science"))

Date: 08/04/2023; 120,636 document results

## CINAHL

**Hyperlink:** <https://search.ebscohost.com/login.aspx?direct=true&AuthType=sso&db=rzh&bquery=co-creat*+OR+co-production+OR+(+public+and+patient+involvement+)+OR+public+participation+OR+participatory+OR+experience+based+design+OR+co-design+OR+user+involvement+OR+collaborative+design+OR+citizen+science&cli0=AA1&clv0=Y&cli1=DT1&clv1=202112-202304&cli2=LA1&clv2=Y&cli3=RV&clv3=Y&authtype=sso&custid=s8849815&type=1&searchMode=Standard&site=ehost-live&scope=site>

**Full Search Field:** co-creat* OR co-production OR ( public and patient involvement ) OR public participation OR participatory OR experience based design OR co-design OR user involvement OR collaborative design OR citizen science

Date: (09/04/2023) ; 2,298 document results

## Pubmed:

("co creat*"[Title/Abstract] OR "co-production"[Title/Abstract] OR "public and patient involvement"[Title/Abstract] OR "public participation"[Title/Abstract] OR "Participatory"[Title/Abstract] OR "experience based design"[Title/Abstract] OR "co-design"[Title/Abstract] OR "user involvement"[Title/Abstract] OR "collaborative design"[Title/Abstract] OR "citizen science"[Title/Abstract]) AND ((booksdocs[Filter] OR casereports[Filter] OR classicalarticle[Filter] OR clinicalstudy[Filter] OR clinicaltrial[Filter] OR clinicaltrialprotocol[Filter] OR clinicaltrialphasei[Filter] OR clinicaltrialphaseii[Filter] OR clinicaltrialphaseiii[Filter] OR clinicaltrialphaseiv[Filter] OR veterinaryclinicaltrial[Filter] OR comparativestudy[Filter] OR correctedandrepublishedarticle[Filter] OR dataset[Filter] OR editorial[Filter] OR electronicsupplementarymaterials[Filter] OR evaluationstudy[Filter] OR festschrift[Filter] OR governmentpublication[Filter] OR guideline[Filter] OR historicalarticle[Filter] OR introductoryjournalarticle[Filter] OR journalarticle[Filter] OR meta-analysis[Filter] OR observationalstudy[Filter] OR veterinaryobservationalstudy[Filter] OR overall[Filter] OR practiceguideline[Filter] OR pragmaticclinicaltrial[Filter] OR randomizedcontrolledtrial[Filter] OR researchsupportamericanrecoveryandreinvestmentact[Filter] OR researchsupportnihextramural[Filter] OR researchsupportnihintramural[Filter] OR researchsupportnonusgovt[Filter] OR researchsupportusgovtnonphs[Filter] OR researchsupportusgovtphs[Filter] OR researchsupportusgovernment[Filter] OR review[Filter] OR scientificintegrityreview[Filter] OR systematicreview[Filter] OR technicalreport[Filter] OR twinstudy[Filter] OR validationstudy[Filter]) AND (humans[Filter] OR animal[Filter]) AND (1970/1/1:3000/12/12[pdat]) AND (english[Filter]))

Date: 10/04/2023; 3,149 document results

## ProQuest

**Full Search Field:** (noft(co-creat*) OR noft(co-conception) OR noft(co-production) OR noft("public and patient involvement") OR noft("public participation") OR noft(participatory) OR noft("experience based design") OR noft(co-design) OR noft("user involvement") OR noft("collaborative design") OR noft("citizen science")) AND (at.exact(("Article" OR "Feature" OR "Report" OR "Undefined" OR "Book" OR "Review" OR "Book Chapter" OR "Editorial" OR "General Information" OR "Case Study" OR "Reference Document" OR "Evidence Based Healthcare" OR "Literature Review" OR "Instructional Material/Guideline" OR "Government & Official Document" OR "Statistics/Data Report" OR "Letter to the Editor" OR "Industry Report" OR "Technical Report") NOT ("News" OR "Commentary" OR "Conference" OR "Conference Proceeding" OR "Speech/Lecture" OR "Correspondence" OR "Interview" OR "Correction/Retraction" OR "Front Page/Cover Story" OR "Bibliography" OR "Transcript" OR "Company Profile" OR "Dissertation/Thesis" OR "Working Paper/Pre-Print" OR "Obituary" OR "Biography" OR "Business Case" OR "Website/Webcast" OR "Financial Materials" OR "Conference Paper" OR "Front Matter" OR "Memoir/Personal Document" OR "Directory" OR "Market Research" OR "Blog" OR "Credit/Acknowledgement" OR "Fiction" OR "Poem" OR "Image/Photograph" OR "Prose" OR "Translation" OR "Back Matter" OR "Essay" OR "Fund/Grant/Fellowship/Award" OR "Editorial Cartoon/Comic" OR "Illustration" OR "Pamphlet/Ephemera" OR "Play" OR "Recipe" OR "Standard" OR "Table Of Contents" OR "Table of Contents")) AND stype.exact(("Scholarly Journals" OR "Wire Feeds" OR "Trade Journals" OR "Books" OR "Reports" OR "Other Sources" OR "Government & Official Publications") NOT ("Newspapers" OR "Dissertations & Theses" OR "Conference Papers & Proceedings" OR "Magazines" OR "Working Papers" OR "Blogs, Podcasts, & Websites" OR "Encyclopedias & Reference Works" OR "Speeches & Presentations" OR "Audio & Video Works")) AND la.exact("ENG") AND pd(19700101-20221231) AND PEER(yes))

Date: 29/03/2023; 11,173 document results

# 2. Training of the classification model

To be able to find the relevant reference within our new set, we trained a classification machine learning model based on BERT [1]. We used the data obtained during the process to create version 1.5 as training data. It was composed of 13,501 relevant and 11,281 irrelevant references (double screener exclusion).

Different combinations of models and features have been tested [2]. We chose the model that reduces the rate of false negatives as much as possible. The chosen configuration gave us the following quality metrics:

- Accuracy: 0.684490399
- f1: 0.731117825
- Precision: 0.635726795
- Recall: 0.860189573

Using this model, we can expect that it will find 86.019% of the relevant papers (i.e., a false negative rate of 13.981%) including a noise of 36.427% false positives.

# 3. Step of the process

Table 1 below represents the different steps of data treatment.

*Table 1. Steps off the data treatment process*

| Database | Step 1: Search results | Step 2: Removal of missing abstracts | Step 3: Duplicates removal (within each set) | Step 4: Selection by the model (in total) | Step 5: Duplicates removal (in total) | Step 6: Comparison with references treated to do version 1.5 |
| --- | --- | --- | --- | --- | --- | --- |
| **Scopus** | 153656 | 150102 | 137554 |  |  |  |
| **Web of Science** | 120636 | 114467 | 113516 |  |  |  |
| **ProQuest** | 11173 | 11121 | 9359 |  |  |  |
| **PubMed** | 3149 | 3107 | 3107 |  |  |  |
| **CINAHL** | 2298 | 2298 | 2261 |  |  |  |
| Total | 290912 | 281095 | 265797 | 73560 | 44029 | 39320 |

Step 1 represents the results for each database. During step 2, we removed the duplicates within each dataset based on an exact match of the title, abstract, or DOI. For step 3, we removed all references without an abstract. We justified this choice because the classification model used title and abstract to define the probability of a reference being relevant. Without an abstract, it reduces importantly the data to be used by the model the estimate the relevancy. After this step, we aggregated all the data. In step 4, we used the model to estimate the references which are relevant based on the training provided with version 1.5. For step 5, we removed the remaining duplicates between databases again on the strict title, abstract, and DOI matches. Finally, during step 6, we removed all references that have been already treated during the creation of version 1.5. To do this, we compared our results with the original search results of version 1.5.

# 4. Accessibility and usability of the databases

The databases are accessible on Zenodo. They mainly contain the title, abstract, DOI, and authors. Two versions are available:

- **Version 1.5** ([DOI: 10.5281/zenodo.6773028](https://doi.org/10.5281/zenodo.7849501)): available in RIS (Research Information Systems) format and CSV (CSV UTF-8). *Quality metrics: 9.38% false negatives; 20.35% false positives.*
- **Version 2.0** ([DOI: 10.5281/zenodo.7849501](https://doi.org/10.5281/zenodo.6773028)): two CSV (CSV UTF-8) files are available. The "Co-Creation Database v2.0 - full.csv" combines the last version, 1.5 and the update, with 52,821 references. The file "Co-Creation Database v2.0 - adding.csv" has only the update, with 39,219 references. *Quality metrics: 13.98% false negatives; ar. 36.43% false positives.*

To perform your search, we recommend you extend your search to the title and abstract since some data are initially missing. The RIS file can be uploaded to any reference manager (e.g., Zotero, Mendeley, etc.) where you will have the feature to search. Concerning CSV files, you can use a Python script to run a Boolean search. You will find one at the following repository: <https://github.com/q5loisel/CCDB-search-python-script.git>

To improve the database in further updates, we make available an online form to submit any irrelevant references you may find while using the last version of the database or to submit any relevant reference not inside the last version. The form is available at the following link: <https://forms.office.com/e/6vu9X0kBcw>.

# References

1. Aum S, Choe S. srBERT: automatic article classification model for systematic review using BERT. *Syst Rev*. 2021;10(1):285. doi:10.1186/s13643-021-01763-w

2. Devlin J, Chang MW, Lee K, Toutanova K. BERT: Pre-training of Deep Bidirectional Transformers for Language Understanding. Published online 2018. doi:10.48550/ARXIV.1810.04805
